# Slow or sudden: Re-interpreting the learning curve for modern systems neuroscience

**DOI:** 10.1016/j.ibneur.2022.05.006

**Published:** 2022-05-24

**Authors:** Sharlen Moore, Kishore V. Kuchibhotla

**Affiliations:** a Department of Psychological and Brain Sciences, Krieger School of Arts and Sciences, Johns Hopkins University, Baltimore, MD, USA; b The Solomon H. Snyder Department of Neuroscience, School of Medicine, Johns Hopkins University, Baltimore, MD, USA; c Department of Biomedical Engineering, School of Engineering, Johns Hopkins University, Baltimore, MD, USA

**Keywords:** Learning, Instrumental learning, Behavior, Systems neuroscience, Large-scale recordings, Big data, Goal-directed learning, circuit, Stimulus-response, Acquisition

## Abstract

Learning is fundamental to animal survival. Animals must learn to link sensory cues in the environment to actions that lead to reward or avoid punishment. Rapid learning can then be highly adaptive and the difference between life or death. To explore the neural dynamics and circuits that underlie learning, however, has typically required the use of laboratory paradigms with tight control of stimuli, action sets, and outcomes. Learning curves in such reward-based tasks are reported as slow and gradual, with animals often taking hundreds to thousands of trials to reach expert performance. The slow, highly variable, and incremental learning curve remains the largely unchallenged belief in modern systems neuroscience. Here, we provide historical and contemporary evidence that instrumental forms of reward-learning can be dissociated into two parallel processes: knowledge acquisition which is rapid with step-like improvements, and behavioral expression which is slower and more variable. We further propose that this conceptual distinction may allow us to isolate the associative (knowledge-related) and non-associative (performance-related) components that influence learning. We then discuss the implications that this revised understanding of the learning curve has for systems neuroscience.

## Introduction

Modern systems neuroscience is going through a methodological revolution that now provides unprecedented access to neural computations during behavior. Large-scale neural recordings, optogenetic perturbation of molecularly-defined circuit elements, and sophisticated computational approaches are being used to reveal how the brain begets behavior—a fundamental goal of neuroscience (Gomez-Marin et al., 2014, Krakauer et al., 2017, Sejnowski et al., 2014). These cutting-edge tools and expanding behavioral repertoires go hand-in-hand as drivers of conceptual and technical innovation in the field.

One particularly holy grail for neuroscience is the ability to understand how neural activity evolves during learning and the underlying circuits that are causally involved. Here, we focus on one area of learning – reward-based instrumental conditioning, a form of associative learning. ‘Instrumental’ (Skinner, 1938) refers to the formation of an association between a behavior and its consequence and it requires the presence of reinforcement (Colwill and Rescorla, 1986, Dickinson, 1994, Staddon and Cerutti, 2003). Traditionally, instrumental forms of learning focus on the relationship between a behavioral response (R) and a biologically relevant outcome (O). Behaviors, however, often occur in the presence of, or are preceded by, stimuli (S) that signal the relevant outcomes. The relationship between stimuli, behaviors, and outcomes (S-R-O) blends stimulus and response learning (e.g., S signals the R-O relationship, S is directly connected to R) (Herrnstein, 1970, Thorndike, 1905, Tolman, 1948). While this framework has evolved over the past 100 years, the core idea that the brain can be understood through learned behaviors (versus reflexes, inaccessible mental processes, or introspection) motivates much of systems neuroscience today. Some of these learned behaviors have been empirically observed to rise rapidly (e.g., conditioned fear) (Blanchard and Blanchard, 1969, Maren, 2001), nevertheless, the formation of reward-based instrumental associations has historically been described as a slow, gradual process despite evidence that there may be faster, step-like improvements (Gallistel et al., 2004). As we will discuss, how we conceptualize the speed of learning, however, has major implications for our understanding of the nature of associative formation and the underlying neural code. A comprehensive review of animal learning theory is beyond the scope of this mini-review but has been covered elsewhere (Bouton, 2016).

## Slow or sudden: empirical observations and interpretation

**Fig. 1:**
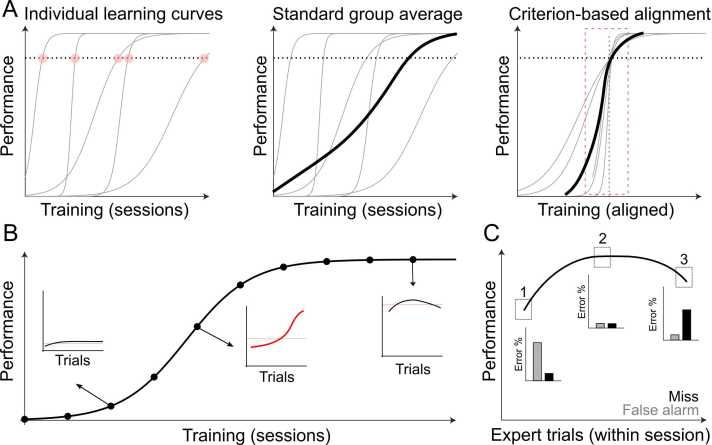
**Methodological drivers of a slow learning curve**. A) The effect of group averaging across animals. *Left,* schematic of individual animal learning curves (gray lines), defined learning criterion (dotted line), and threshold crossings (red circles). *Middle,* averaging individual learning curves aligned to the start of training creates the appearance of a slow and gradual process. *Right,* aligning learning curves to a defined learning criterion identifies a more rapid, and shared, dynamic across animals (within the red dotted box) and may provide better group averaging for use in neural data analysis. B) The effect of session averaging within an animal. Schematic of learning curve across training sessions shows a smooth gradual increase in performance. Early (left inset) and late (right inset) in learning, the session averaged performance provides a reasonable description of the behavior. At the ‘slope’ of the learning curve, however, the within day change (middle inset) can be dramatic with fast transitions in performance that are obscured by session-based averaging. C) The effect of motivation on within day performance. Expert performance can be influenced by an animals’ internal state. Motivation can change over the course of an expert session, driving errors typically ascribed to perceptual judgements. Early in the session (1), over motivation might be the driver of a high false alarm rate, while by the end, satiety might drive an animal to miss. (For interpretation of the references to color in this figure legend, the reader is referred to the web version of this article.)

Early studies of discrimination learning focused on individual animals while also exploring behavior before asymptotic performance, sometimes referred to as the ‘pre-solution’ period. This debate centered on whether animals were engaging in ‘trial-and-error’ learning (Spence, 1936, Spence, 1945) or were, instead, testing ‘hypotheses’ (Krechevsky, 1932a, Lashley, 1929) during this pre-solution period. This question endures but has been understudied as the majority of learning research quickly moved away from individual-centered analysis and towards higher throughput approaches in small animals. This latter shift in approach has led to thinking of instrumental learning as a slow, gradual process with high inter-subject variability. There were at least three methodological drivers of this observation. First, individual animals were grouped and learning curves were averaged. The challenges with group averaging were noted as early as the 1930's, with observations from Krechevsky: *“[…] real and valid information in reference to the behavior of organisms can be obtained only by studying the actual individual as an individual* […]” (Krechevsky, 1932b). This topic was resumed by Estes in the 1950’s (Estes, 1956) and then explicitly analyzed nearly 50 years later (Gallistel et al., 2004, Papachristos and Gallistel, 2006). Group averaging across animals masks the variety of individual learning speeds and obscures the rapidity by which many animals transition from naïve to expert (Fig. 1A). Second, even within individual animals, analytical approaches favored temporal smoothing, binning or fitting across trials. The simplest of these—averaging performance within a session—became modus operandi in behavioral literature and continues to dominate the analysis of learning speeds (Guo et al., 2014). Rapid performance improvements within a session, as those observed in (Arican et al., 2019, de Hoz and Nelken, 2014, Gutierrez et al., 2010, International Brain Laboratory et al., 2021, Komiyama et al., 2010, Mazziotti et al., 2020, Rosenberg et al., 2021, Stoilova et al., 2019), became obscured (Gallistel et al., 2004) and thus, understudied (Fig. 1B). Third, laboratory animals have been put on water or food restriction protocols with externally driven trial schedules (Goltstein et al., 2018, Guo et al., 2014), despite early concerns that thirst is an ‘arbitrary drive’ (Skinner, 1936). The modern approach of both metabolic restriction and fixed trial scheduling has likely led to a ‘ceiling effect’ of over-motivation early in a session and a ‘floor effect’ of under-motivation late in a session (Berditchevskaia et al., 2016, Groblewski et al., 2020, van Swieten and Bogacz, 2020) (Fig. 1C). When combined with temporal smoothing within a session, these ‘non-learning’ effects may cloud learning-related changes. Furthermore, excessive motivation early in a session may impact the animal’s behavioral strategy – incentivizing exploratory errors in impoverished environments. In fact, recent studies demonstrate how ‘errors’ in a rodent decision-making task are more likely due to exploratory strategies than lapses in judgement (Ashwood et al., 2022, Carandini and Churchland, 2013, Pisupati et al., 2021).

These three factors (Fig. 1) have conspired to paint a picture of instrumental learning as slow and variable. This is not to say that the field has been blind to this issue; rather, the purpose of many learning studies, particularly those interested in neural mechanisms, has motivated these approaches. For example, lesion or mutation studies aim to isolate the brain regions involved in learning, and thus necessitate group comparisons (Bey et al., 2018; Cheung and Cardinal, 2005; Corbit et al., 2001, Corbit et al., 2003; Featherstone and McDonald, 2004; Lintas et al., 2021). The desire for reproducibility and reduced variability in such comparisons has likely driven the usage of group and session-based averaging of the learning curves. With that said, deciphering the *neural code* underlying the formation of associations, will require a more nuanced view of learning within individual animals linking trial-by-trial fluctuations in neural activity with behavioral performance. Pinpointing the precise timing of *when animals learn the task contingencies* will be crucial as we aim to identify its neural basis. The low-pass filtering of behavioral performance during learning may inadvertently focus neural interrogations on mechanisms unrelated to core contingency learning.

### Sudden and slow: distinct timescales for acquisition and expression of instrumental learning

In most studies, performance is measured during instrumental learning when reinforcement is available. Reinforcers and rewards can lead to a variety of paradoxical effects. One such effect was initially referred to as a ‘frustration’ response (Amsel and Roussel, 1952, Wagner, 1959). When expert rats trained to run a double runway for a water reward are exposed to reward omission, they surprisingly start running faster (Amsel and Roussel, 1952). Thus, a non-reinforced trial seemed to strengthen the instrumental action. Non-reinforced trials have also played an important role in other forms of learning – notably, fear conditioning, where ‘test’ trials in the absence of the reinforcer (no shock) are the standard way to measure whether a conditioned stimulus has gained control of a freezing response (Britton et al., 2014). Non-reinforced trials, rarely used during reward-based learning, may hold a key to unlocking the true learning curve.

**Fig. 2:**
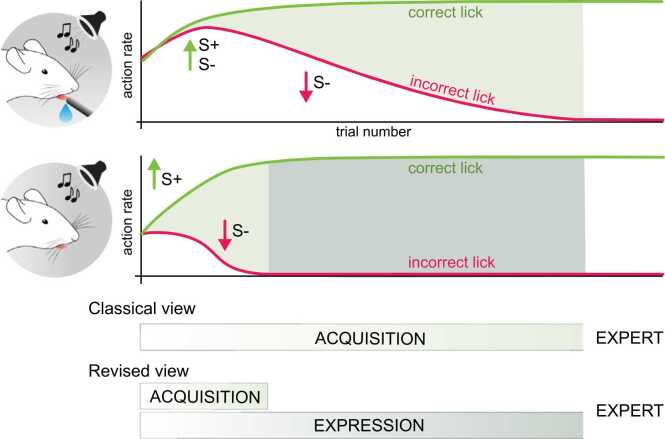
**Behavioral dissociation of acquisition and expression**. Mice were trained on an auditory go/no-go task in which they learn to lick to tone for a water reward (S+) and withhold licking to another tone to avoid a timeout (S-). Performance during learning in a reinforced context (*top*) has classically been equated to the ‘acquisition’ of task contingencies. In our data, we observe similar gradual acquisition trajectories in the reinforced context (top). We unmasked a more rapid acquisition trajectory by removing access to reinforcement in a few trials (bottom), and argue for a second dissociable process, ‘expression’, which reveals learned discriminations.

Recently, we reasoned that non-reinforced trials would provide a more juridical measurement of the acquisition of task contingencies if interleaved during behavioral training (Kuchibhotla et al., 2019). We trained head-fixed mice to respond to one tone (S+) for a water reward and withhold responding to another (S-) to avoid a timeout. We interleaved reinforced trials with those without available reinforcement (‘probe’ trials). Surprisingly, early in learning, animals discriminated between S+ and S- better in probe trials than in reinforced trials. Thus, this task design unmasked the acquisition phase of S+ and S- discrimination learning, shown only in probe trials, that occurred quickly and was stereotyped across animals. This underlying learned discrimination was then revealed during reinforced trials in a slower, more variable phase, termed ‘expression’ (Fig. 2). We expanded our studies to freely moving rats and head-fixed ferrets and found a nearly identical distinction across a wide range of tasks, including Pavlovian, instrumental, and occasion setting tasks (Kuchibhotla et al., 2019). These experiments provide evidence supporting a learning framework in which there are two parallel learning processes: one more rapid and stereotyped (the core contingency learning, *acquisition*) and one slower and more variable (*expression*). One subtlety that arises is that assaying task knowledge in non-reinforced probe trials still relies on a behavioral output that is learned when the reinforcer is available. Regardless, the implication of this study for the timing of associative learning is clear: the contingencies are learned early and lead to rapid improvements within a tight temporal window. Performance in non-reinforced trials, in turn, provides a practical tool for criterion-based alignment (**Fig. 1A**, right) to more precisely link behavior during learning with its underlying neural drivers.

Another implication is that animal performance during learning can sometimes mask their underlying knowledge. Behavioral expression in the presence of reinforcement (performance) may reflect other factors, including exploration or over-motivation, that obfuscate the measurement of the learned association (knowledge). This dissociation between knowledge and performance relates to a classic distinction made in experimental psychology and linguistics, which differentiates the performance of a system from its underlying competence (Chomsky, 1969, Feigenson et al., 2004, Spelke et al., 1992). Put more simply, what you know can be very different from what you show*.* For example, infants do not tend to reach for hidden objects until they are ~8 months old (Baillargeon et al., 1990), leading Piaget to infer that younger infants lack object permanence: they do not know that objects continue to exist when they are hidden (Piaget, 1954). Pioneering studies, however, exploited the discovery that infants will look longer at events that are surprising (Stahl and Feigenson, 2015). They demonstrated that if an object is hidden by an occluder and subsequently the occluder is lifted and the object is now gone, infants will look longer at this surprising disappearance (Baillargeon et al., 1985). This revealed a hidden competence at 5-months of age that was masked by a motor confound in Piaget’s original studies.

Here, we argue that animals exhibit a similar distinction between performance and competence during learning. Competence reflects the animal’s underlying knowledge of the task contingencies. Performance, on the other hand, refers to how animals express their knowledge and is subject to non-associative factors that may relate to internal state or external context. We argue that to uncover the neural basis of learning requires re-interpreting the learning curve as incorporating both processes.

## Unlocking the neural code for instrumental learning

The advent of large-scale neural recordings and manipulation techniques during learning opens up the possibility to determine exactly how the neural circuits form associations. To do so, we need to overcome at least two major challenges. One is the difficulty of gaining access to an animal’s core task knowledge during learning, which first requires to behaviorally identify *when* the knowledge is acquired versus expressed. Another is the challenge of catching a moving target: the brain and behavior are ‘ever-changing’ during learning. The possibility that the associative aspects of learning occur more quickly than previously thought has major implications for how we link learning processes with neural activity. Here we outline a framework for understanding neural data acquired during learning with the expressed intent of addressing the above challenges and avoiding misinterpretations due to biases in our analytical methods.

### Dissociating knowledge from performance using multi-dimensional behavioral metrics

During learning, decision-making processes are in flux and are not only influenced by changes in associative strength between stimuli, actions, and reinforcers but can also be influenced by changes in behavioral strategy, internal state, or external context. Standard approaches of using categorical outcomes (correct vs incorrect, hit vs miss) or binary action variables (go vs no-go, left vs right) may not allow for a distinction between the associative and non-associative influences on the decision process. This realization over the past decade has led to major shifts in our thinking of decision-making *after learning.* Emerging studies have used detailed analysis of behavioral microstructures to demonstrate that animals show different strategies based on hedonic state (Dwyer, 2012, Johnson et al., 2010) or exploratory drive (Luksys et al., 2009, Pisupati et al., 2021) and exhibit different types of errors based on their level of arousal (de Gee et al., 2014, de Gee et al., 2020) or motivation (Berditchevskaia et al., 2016, Groblewski et al., 2020). For example, in expert animals, it is possible to identify structured changes in performance as a function of motivation (Berditchevskaia et al., 2016) (Fig. 1C). Early in an expert session during a go/no-go task, water-restricted animals will tend to increase false alarms (responding to the S-) due to excessive motivation. Late in the same session, satiated animals will begin to reduce responding to the S+ (miss). These errors are not related to a perceptual judgement but are instead due to factors influenced by their internal state (Berditchevskaia et al., 2016, Groblewski et al., 2020). Such differences—though demonstrated in expert animals—likely serve as confounds for association formation during learning. Using novel approaches with the potential to modulate motivation (Reinagel, 2018, Urai et al., 2021) and more detailed behavior measurements, including movement (Musall et al., 2019, Salkoff et al., 2020, Stringer et al., 2019), pupil fluctuation (de Gee et al., 2014, de Gee et al., 2020), and orofacial movements (Bollu et al., 2021, Dolensek et al., 2020), will allow us to infer the animal’s state throughout the learning process and better identify the non-associative factors that influence performance during learning.

Here, we argue that a detailed analysis of the *evolution of behavioral microstructures* will be critical to dissociate associative components of learning (i.e. knowledge) from non-associative factors that may influence performance. To better isolate the formation of associations will also require moving beyond the binary categories in action or outcome variables. In the auditory go/no-go task described in Fig. 2, for example, a major component of discriminative learning is the ability for mice to withhold licking to the S-. Measuring response latency and response vigor on false alarm trials surprisingly reveals that animals begin to delay licking to the S- (longer lick latency) much earlier than if measured only as a categorical variable. Thus, by shifting from a ‘digital’ readout (lick vs. no lick) to an ‘analog’ readout (latency and vigor), we can identify behavioral correlates of associative formation that provide a better temporal window for identifying neural drivers. Integrating these analog measures of behavior during learning, with more standard digital measures of action outcomes, will be essential to identify exactly when associations begin forming and the underlying neural implementation.

### Catching a moving target: trial-by-trial alignment of behavioral and neural data

We detailed above how group averaging produces slow, gradual learning curves despite evidence that individual animals often learn quickly, showing step-like improvements at discrete timepoints (Fig. 1A). Group averaging, however, offers major advantages when considering neural data as it provides an analytical approach to identify common neural processes across animals while reducing the possibility of spurious correlations. How can we account for individual differences in learning rate while also allowing for group averaging? To date, the most common way of averaging cohorts is aligning all animals to the onset of training. The onset of training, however, is defined by the experimenter rather than the underlying behavioral learning process used by the animal. To circumvent this, one possibility is to (1) identify key behavioral indicators of learning (e.g., trial block when performance reaches a criterion) and then (2) align animal learning trajectories based on these criteria (Fig. 1A). This criterion-based approach to alignment and group averaging will allow the *behavior* to drive the neural data analysis and has already proven valuable in understanding learning-related dynamics in the somatosensory cortex of mice (Chen et al., 2015). More broadly, behavioral evidence of learning may not directly correlate with when associations are formed, but rather, provides a cutoff before which the associative processes may occur. By aligning behavioral data across animals that focuses on the learning process, it may be possible to uncover shared activity patterns across animals that point to common neurobiological mechanisms. The goal of dissociating the associative and non-associative components of learning will also be served by more advanced computational approaches of interpreting neural data on a trial-by-trial level and distinguishing single-neuron activity profiles from population codes.

## Outlook: constraining big neural data with a revised conceptual model of instrumental learning

We have provided evidence that core contingency learning may occur more rapidly than previously thought, with improvements happening within a few dozens of trials (Kuchibhotla et al., 2019). Averaging trials, either across full sessions or in large trial bins, may obscure the neural activity changes that occur at precise timepoints that subserve the associative learning process. Synthetic trial-by-trial approaches are now emerging that combine large-scale neural data acquisition with computational approaches that can be constrained by model-based predictions (Steinmetz et al., 2019, Urai et al., 2022). In addition, recent work that aims to explain trial-by-trial variability through the lens of changes in internal states will be a valuable guide as we try to pinpoint the neural processes related to behavioral expression on a slower and more variable timescale. Some of the heterogeneity in neural activity may reflect ongoing changes in performance-related (rather than knowledge-related) computations and these changes can be inferred by relating neural activity to ongoing changes in behavioral microstructures, including spontaneous movements. Computational modeling will be critical to distinguish between *knowledge* and *performance* drivers of neural activity. Descriptive models (Ashwood et al., 2022, Deliano et al., 2016, Roy et al., 2021) may help identify drivers of performance variability during learning that reflect distinct strategies or motivational levels. In addition, normative decision-theoretic models (Dayan and Daw, 2008, Dayan and Niv, 2008, Niv et al., 2006, Pisupati et al., 2021, Rao, 2010) will help separate associative, policy-level and read-out computations underlying the dissociable components of behavioral learning.

Large-scale neural recordings provide an opportunity to better understand how the brain implements a variety of critical behavioral computations, including instrumental learning. Here, we argue that re-visiting our understanding of the *shape* of the learning curve and its underlying cognitive drivers is essential to interpreting big neural data. Rather than thinking about learning as either ‘slow’ or ‘sudden’; we argue that learning is better interpreted as a combination of the two. We provide evidence that instrumental forms of reward-learning can be dissociated into two parallel processes: knowledge acquisition which is rapid with step-like improvements and behavioral expression which is slower and more variable. We further propose that this conceptual distinction may allow us to isolate the associative (knowledge-related) and non-associative (performance-related) components that influence learning. The core idea, that underlying knowledge and the use of that knowledge, are distinct has been paralleled in experimental psychology and linguistics—famously introduced by Chomsky over 60 years ago (Chomsky, 1969). In an era of big neural data—where recording from thousands of neurons, across multiple brain regions and over many days is no longer a dream but a reality—it will be important to be guided by a rich behavioral understanding of *how* and *when* animals acquire and then express task knowledge.

## References

[bib1] Amsel A., Roussel J. (1952). Motivational properties of frustration. I. Effect on a running response of the addition of frustration to the motivational complex. J. Exp. Psychol..

[bib2] Arican C., Bulk J., Deisig N., Nawrot M.P. (2019). Cockroaches show individuality in learning and memory during classical and operant conditioning. Front. Physiol..

[bib3] Ashwood, Z.C., Roy, N.A., Stone, I.R., International Brain Laboratory, Urai, A.E., Churchland, A.K., Pouget, A., and Pillow, J.W. (2022). Mice alternate between discrete strategies during perceptual decision-making. Nat. Neurosci. *25*, 201–212. 10.1038/s41593-021-01007-z PMC8890994 35132235

[bib4] Baillargeon R., Spelke E.S., Wasserman S. (1985). Object permanence in five-month-old infants. Cognition.

[bib5] Baillargeon R., Graber M., Devos J., Black J. (1990). Why do young infants fail to search for hidden objects?. Cognition.

[bib6] Berditchevskaia A., Cazé R.D., Schultz S.R. (2016). Performance in a GO/NOGO perceptual task reflects a balance between impulsive and instrumental components of behaviour. Sci. Rep..

[bib7] Bey A.L., Wang X., Yan H., Kim N., Passman R.L., Yang Y., Cao X., Towers A.J., Hulbert S.W., Duffney L.J. (2018). Brain region-specific disruption of Shank3 in mice reveals a dissociation for cortical and striatal circuits in autism-related behaviors. Transl. Psychiatry.

[bib8] Blanchard R.J., Blanchard D.C. (1969). Passive and active reactions to fear-eliciting stimuli. J. Comp. Physiol. Psychol..

[bib9] Bollu T., Ito B.S., Whitehead S.C., Kardon B., Redd J., Liu M.H., Goldberg J.H. (2021). Cortex-dependent corrections as the tongue reaches for and misses targets. Nature.

[bib10] Bouton M.E. (2016). Synthesis.

[bib11] Britton J.C., Evans T.C., Hernandez M.V. (2014). Looking beyond fear and extinction learning: considering novel treatment targets for anxiety. Curr. Behav. Neurosci. Rep..

[bib12] Carandini M., Churchland A.K. (2013). Probing perceptual decisions in rodents. Nat. Neurosci..

[bib13] Chen J.L., Margolis D.J., Stankov A., Sumanovski L.T., Schneider B.L., Helmchen F. (2015). Pathway-specific reorganization of projection neurons in somatosensory cortex during learning. Nat. Neurosci..

[bib14] Cheung T.H.C., Cardinal R.N. (2005). Hippocampal lesions facilitate instrumental learning with delayed reinforcement but induce impulsive choice in rats. BMC Neurosci..

[bib15] Chomsky, N.,1969. Aspects of the Theory of Syntax (M.I.T. Press).

[bib16] Colwill R.M., Rescorla R.A. (1986). In Psychology of Learning and Motivation.

[bib17] Corbit L.H., Muir J.L., Balleine B.W. (2001). The role of the nucleus accumbens in instrumental conditioning: evidence of a functional dissociation between accumbens core and shell. J. Neurosci..

[bib18] Corbit L.H., Muir J.L., Balleine B.W. (2003). Lesions of mediodorsal thalamus and anterior thalamic nuclei produce dissociable effects on instrumental conditioning in rats. Eur. J. Neurosci..

[bib19] Dayan P., Daw N.D. (2008). Decision theory, reinforcement learning, and the brain. Cogn. Affect Behav. Neurosci..

[bib20] Dayan P., Niv Y. (2008). Reinforcement learning: the good, the bad and the ugly. Curr. Opin. Neurobiol..

[bib21] Deliano M., Tabelow K., König R., Polzehl J. (2016). Improving accuracy and temporal resolution of learning curve estimation for within- and across-session analysis. PLoS One.

[bib22] Dickinson A. (1994). In Animal learning and cognition.

[bib23] Dolensek N., Gehrlach D.A., Klein A.S., Gogolla N. (2020). Facial expressions of emotion states and their neuronal correlates in mice. Science.

[bib24] Dwyer D.M. (2012). Licking and liking: the assessment of hedonic responses in rodents. Q J. Exp. Psychol..

[bib25] Estes W.K. (1956). The problem of inference from curves based on group data. Psychol. Bull..

[bib26] Featherstone R.E., McDonald R.J. (2004). Dorsal striatum and stimulus-response learning: lesions of the dorsolateral, but not dorsomedial, striatum impair acquisition of a stimulus-response-based instrumental discrimination task, while sparing conditioned place preference learning. Neuroscience.

[bib27] Feigenson L., Dehaene S., Spelke E. (2004). Core systems of number. Trends Cogn. Sci..

[bib28] Gallistel C.R., Fairhurst S., Balsam P. (2004). The learning curve: implications of a quantitative analysis. Proc. Natl. Acad. Sci. USA.

[bib29] de Gee J.W., Knapen T., Donner T.H. (2014). Decision-related pupil dilation reflects upcoming choice and individual bias. Proc. Natl. Acad. Sci. USA.

[bib30] de Gee J.W., Tsetsos K., Schwabe L., Urai A.E., McCormick D., McGinley M.J., Donner T.H. (2020). Pupil-linked phasic arousal predicts a reduction of choice bias across species and decision domains. Elife.

[bib31] Goltstein P.M., Reinert S., Glas A., Bonhoeffer T., Hübener M. (2018). Food and water restriction lead to differential learning behaviors in a head-fixed two-choice visual discrimination task for mice. PLoS One.

[bib32] Gomez-Marin A., Paton J.J., Kampff A.R., Costa R.M., Mainen Z.F. (2014). Big behavioral data: psychology, ethology and the foundations of neuroscience. Nat. Neurosci..

[bib33] Groblewski P.A., Ollerenshaw D.R., Kiggins J.T., Garrett M.E., Mochizuki C., Casal L., Cross S., Mace K., Swapp J., Manavi S. (2020). Characterization of learning, motivation, and visual perception in five transgenic mouse lines expressing gcamp in distinct cell populations. Front. Behav. Neurosci..

[bib34] Guo Z.V., Hires S.A., Li N., O’Connor D.H., Komiyama T., Ophir E., Huber D., Bonardi C., Morandell K., Gutnisky D. (2014). Procedures for behavioral experiments in head-fixed mice. PLoS One.

[bib35] Gutierrez R., Simon S.A., Nicolelis M.A.L. (2010). Licking-induced synchrony in the taste-reward circuit improves cue discrimination during learning. J. Neurosci..

[bib36] Herrnstein R.J. (1970). On the law of effect. J. Exp. Anal. Behav..

[bib37] de Hoz L., Nelken I. (2014). Frequency tuning in the behaving mouse: different bandwidths for discrimination and generalization. PLoS One.

[bib38] International Brain Laboratory, Aguillon-Rodriguez V., Angelaki D., Bayer H., Bonacchi N., Carandini M., Cazettes F., Chapuis G., Churchland A.K., Dan Y. (2021). Standardized and reproducible measurement of decision-making in mice. Elife.

[bib39] Johnson A.W., Sherwood A., Smith D.R., Wosiski-Kuhn M., Gallagher M., Holland P.C. (2010). An analysis of licking microstructure in three strains of mice. Appetite.

[bib40] Komiyama T., Sato T.R., O’Connor D.H., Zhang Y.-X., Huber D., Hooks B.M., Gabitto M., Svoboda K. (2010). Learning-related fine-scale specificity imaged in motor cortex circuits of behaving mice. Nature.

[bib41] Krakauer J.W., Ghazanfar A.A., Gomez-Marin A., MacIver M.A., Poeppel D. (2017). Neuroscience needs behavior: correcting a reductionist bias. Neuron.

[bib42] Krechevsky I. (1932). Hypotheses" in rats. Psychol. Rev..

[bib43] Krechevsky I. (1932). Hypotheses“ versus” chance in the pre-solution period in sensory discrimination-learning. Univ. Calif. Publ. Psychol..

[bib44] Kuchibhotla K.V., Sten Hindmarsh, Papadoyannis T., Elnozahy E.S., Fogelson S., Kumar K.A., Boubenec R., Holland Y., Ostojic, S P.C., Froemke R.C. (2019). Dissociating task acquisition from expression during learning reveals latent knowledge. Nat. Commun..

[bib45] Lashley K.S. (1929).

[bib46] Lintas A., Sánchez-Campusano R., Villa A.E.P., Gruart A., Delgado-García J.M. (2021). Operant conditioning deficits and modified local field potential activities in parvalbumin-deficient mice. Sci. Rep..

[bib47] Luksys G., Gerstner W., Sandi C. (2009). Stress, genotype and norepinephrine in the prediction of mouse behavior using reinforcement learning. Nat. Neurosci..

[bib48] Maren S. (2001). Neurobiology of Pavlovian fear conditioning. Annu. Rev. Neurosci..

[bib49] Mazziotti R., Sagona G., Lupori L., Martini V., Pizzorusso T. (2020). 3D printable device for automated operant conditioning in the mouse. eNeuro *7*.

[bib50] Musall S., Kaufman M.T., Juavinett A.L., Gluf S., Churchland A.K. (2019). Single-trial neural dynamics are dominated by richly varied movements. Nat. Neurosci..

[bib51] Niv Y., Joel D., Dayan P. (2006). A normative perspective on motivation. Trends Cogn. Sci..

[bib52] Papachristos E.B., Gallistel C.R. (2006). Autoshaped head poking in the mouse: a quantitative analysis of the learning curve. J. Exp. Anal. Behav..

[bib53] Piaget J. (1954).

[bib54] Pisupati S., Chartarifsky-Lynn L., Khanal A., Churchland A.K. (2021). Lapses in perceptual decisions reflect exploration. Elife.

[bib55] Rao R.P.N. (2010). Decision making under uncertainty: a neural model based on partially observable markov decision processes. Front. Comput. Neurosci..

[bib56] Reinagel P. (2018). Training rats using water rewards without water restriction. Front. Behav. Neurosci..

[bib57] Rosenberg M., Zhang T., Perona P., Meister M. (2021). Mice in a labyrinth show rapid learning, sudden insight, and efficient exploration. Elife.

[bib58] Roy N.A., Bak J.H., International Brain Laboratory, Akrami A., Brody C.D., Pillow J.W. (2021). Extracting the dynamics of behavior in sensory decision-making experiments. Neuron.

[bib59] Salkoff D.B., Zagha E., McCarthy E., McCormick D.A. (2020). Movement and performance explain widespread cortical activity in a visual detection task. Cereb. Cortex.

[bib60] Sejnowski T.J., Churchland P.S., Movshon J.A. (2014). Putting big data to good use in neuroscience. Nat. Neurosci..

[bib61] Skinner B.F. (1936). Thirst as an arbitrary drive. J. Gen. Psychol..

[bib62] Skinner B.F. (1938). Analysis.

[bib63] Spelke E.S., Breinlinger K., Macomber J., Jacobson K. (1992). Origins of knowledge. Psychol. Rev..

[bib64] Spence K.W. (1936). The nature of discrimination learning in animals. Psychol. Rev..

[bib65] Spence K.W. (1945). An experimental test of the continuity and non-continuity theories of discrimination learning. J. Exp. Psychol..

[bib66] Staddon J.E.R., Cerutti D.T. (2003). Operant conditioning. Annu. Rev. Psychol..

[bib67] Stahl A.E., Feigenson L. (2015). Cognitive development. Observing the unexpected enhances infants’ learning and exploration. Science.

[bib68] Steinmetz N.A., Zatka-Haas P., Carandini M., Harris K.D. (2019). Distributed coding of choice, action and engagement across the mouse brain. Nature.

[bib69] Stoilova V.V., Wette S.A., Stüttgen M.C. (2019). A free-operant reward-tracking paradigm to study neural mechanisms and neurochemical modulation of adaptive behavior in rats. Int. J. Mol. Sci..

[bib70] Stringer C., Pachitariu M., Steinmetz N., Reddy C.B., Carandini M., Harris K.D. (2019). Spontaneous behaviors drive multidimensional, brainwide activity. Science.

[bib71] Thorndike E.L. (1905).

[bib72] Tolman E.C. (1948). Cognitive maps in rats and men. Psychol. Rev..

[bib73] Urai A.E., Aguillon-Rodriguez V., Laranjeira I.C., Cazettes F., International Brain Laboratory, Mainen Z.F., Churchland A.K. (2021). Citric acid water as an alternative to water restriction for high-yield mouse behavior. eNeuro.

[bib74] Urai A.E., Doiron B., Leifer A.M., Churchland A.K. (2022). Large-scale neural recordings call for new insights to link brain and behavior. Nat. Neurosci..

[bib75] van Swieten M.M.H., Bogacz R. (2020). Modeling the effects of motivation on choice and learning in the basal ganglia. PLoS Comput. Biol..

[bib76] Wagner A.R. (1959). The role of reinforcement and nonreinforcement in an apparent frustration effect. J. Exp. Psychol..

